# Bone Marrow Mesenchymal Stem Cells Inhibit the Function of Dendritic Cells by Secreting Galectin-1

**DOI:** 10.1155/2017/3248605

**Published:** 2017-06-21

**Authors:** Yue Zhang, Xia-hui Ge, Xue-Jun Guo, Si-bin Guan, Xiao-ming Li, Wen Gu, Wei-guo Xu

**Affiliations:** Department of Respiratory Medicine, Xinhua Hospital Affiliated to Shanghai Jiao Tong University School of Medicine, Shanghai 200092, China

## Abstract

This study aimed to investigate whether bone marrow-derived mesenchymal stem cells (BM-MSCs) can inhibit function of dendritic cells (DCs) by secreting Galectin-1 (Gal-1). BM-MSCs have been shown to inhibit the maturation and function of DCs, further inhibiting the activation and proliferation of T cells. However, the detailed mechanism remains unknown. In this current study, MSCs and DCs derived from mouse bone marrow were cocultured using Transwell culture plates under different in vitro conditions. The results showed that as the ratio of MSC to DC of the coculture system increased and the coculture time of the two cells prolonged, the concentrations of Gal-1, interleukin- (IL-) 10, and IL-12 in the supernatants were increased and the protein expression of Gal-1 on and within DCs was also enhanced. The phosphorylation of extracellular signal-regulated kinase (ERK) pathway in DCs was boosted, whereas p38 mitogen-activated protein kinase (MAPK) pathway phosphorylation was weakened. Meanwhile, the expression of costimulatory molecules on the surface of DCs was decreased, and the proliferative effect of DCs on allogeneic T cells was also decreased. Therefore, this present study indicated that Gal-1 secreted from MSCs upregulated expression of Gal-1 and stimulated formation of tolerance immunophenotype on DCs, where the underlying mechanism was the regulation of the MAPK signaling pathway in DCs, thereby inhibiting the function of DCs.

## 1. Introduction

Bone marrow-derived mesenchymal stem cells (BM-MSCs) are a class of pluripotent stem cells with potent proliferative, self-renewing, and pluripotent properties. They have been extensively studied over the past decade due to their low immunogenicity and a wide range of immunomodulatory effects. MSCs interact with diverse immune cells including macrophages, B cells, natural killer cells, and T cells [1, 2] for their anti-inflammatory and anti-injury effects.

Dendritic cells (DCs) are widely known to be the most powerful full-time antigen-presenting cells. They activate the initial T lymphocytes [3, 4] and play an important role in immune self-stability and graft tolerance. Decreasing expression of CD80, CD83, CD86, and major histocompatibility complex (MHC) II on the surface of DCs can inhibit the proliferative effects on T cells. Recent studies found that MSCs inhibit DC-induced T cell activation and proliferation, thereby inhibiting the body's immune response and promoting the development of immune tolerance [5, 6]. The present study found that coculture of MSCs and DCs in vitro inhibited differentiation, maturation, and activation of DCs, through downregulating the expression of costimulatory molecules on the surface of DCs. This process involves a variety of mechanisms: some studies proposed that [7] MSCs played their inhibitory role through direct contact with DCs, while some other studies reported MSCs inhibited DCs probably by secreting soluble factors [8, 9]. However, detailed mechanisms underlying the inhibitory effects of MSCs on DC functions are still unclear.

Galectin-1 (Gal-1) is the first member discovered in Galectin family, and its expression is induced by a variety of physiological and pathological factors. Studies have shown that Gal-1 inhibits functions of monocytes and macrophages [10], as well as migration of lymphocytes and neutrophils to inflammatory sites [11]. Gal-1 also has immunosuppressive effects. Deák and colleagues found [12] Gal-1 of high concentration in vitro induced the apoptosis of T cells; even a low concentration can promote weakening of T cell–extracellular matrix adhesion, leading to decreased production of proinflammatory factors, including tumor necrosis factor alpha (TNF-*α*) and interferon gamma (IFN-*γ*), and increased production of anti-inflammatory factors [13]. These findings indicated that Gal-1 was an important factor in inhibiting regulatory T cells activity. Recent studies have also discovered that Gal-1 can regulate the differentiation and migration of DC and confer DCs with induced tolerance potential [14, 15]. All these studies suggest that Gal-1 plays an important role in immunomodulatory and immunosuppressive responses.

Mitogen-activated protein kinase (MAPK) is a serine/threonine protein kinase existing in mammals. This kinase regulates cell growth, proliferation, differentiation, transformation, adaptation to environmental stress, inflammatory responses, and many other important cellular physiological/pathological processes. Studies have shown that the MAPK pathway is largely involved in differentiation and maturation of DCs. Whether Gal-1 and MAPK are involved in the immunosuppressive processes of MSCs on DCs is still inconclusive.

Above all, this present study hypothesizes that (1) MSC inhibits the activation and proliferation of allogeneic T cells by secreting Gal-1 and (2) Gal-1 plays an immunosuppressive role through modulating MAPK pathway in DCs.

## 2. Materials and Methods

### 2.1. Ethical Approval

Six-week-old female BALB/c and C57 mice were purchased from Shanghai Laboratory Animal Center Co. (Shanghai, China). All mice were raised strictly according to the National Institutes of Health Guidelines on the Use of Laboratory Animals. In addition, related experimental protocols were carefully designed and approval of the Committee on the Ethics of Animal Care and Use of Chinese People's Liberation Army General Hospital guaranteed the normalization of this study. All surgeries were performed under sodium pentobarbital anesthesia, and all efforts were made to minimize suffering.

### 2.2. MSC Isolation and Culture

Bone marrow was obtained from BALB/c mice bilateral femur and tibia, and the cells were resuspended in 10% fetal bovine serum (FBS) (Gibco, USA) containing Dulbecco's modified Eagle's complete medium/nutrient mixture F-12 (Hyclone, USA; with 1% penicillin–streptomycin), with a cell density of 2 × 10^6^/mL. Resuspended cells were cultured in an incubator at 37°C supplemented with 5% (v/v) CO_2_. The medium was changed once every 48–72 h to gradually purify the cells. When the cell confluency reached 85%–90%, the cells were treated with 0.25% trypsin (Gibco, USA) for digestion and subculture expansion. MSCs were not used in subsequent experiments until after three passages to fully remove monocytes/macrophages.

### 2.3. DCs Isolation and Culture

Similar to BM-MSCs, DCs were also obtained from BALB/c mice bilateral femur and tibia. The cells were seeded in six-well plates and resuspended in Roswell Park Memorial Institute (RPMI) 1640 medium (Hyclone, USA, with 1% penicillin–streptomycin) containing 10% FBS (Gibco, USA), followed by addition of granulocyte macrophage colony-stimulating factor (GM-CSF) (final concentration: 10 ng/mL) and interleukin (IL)-4 (final concentration: 1 ng/mL) (GM-CSF and IL-4 were from PeproTech, UK). The cell density was 2 × 10^6^/mL. Cells were cultured in incubator supplemented with 5% CO_2_ at 37°C. The full volume of the medium was changed at day 3, and half volume at day 5. Sufficient amounts of GM-CSF (final concentration of 10 ng/mL) and IL-4 (final concentration of 1 ng/mL) were supplemented every time during medium change. Suspended cells were collected at day 6.

### 2.4. MSC and DC Transwell Coculture

Pore size of membrane used was 0.4 *μ*m (Corning, USA) so cells can not get through it. DCs were cultured in lower chamber. Transwell culture plates were resuspended using DC complete culture medium containing GM-CSF (final concentration: 10 ng/mL) and IL-4 (final concentration: 1 ng/mL). Each well contained 1 × 10^6^ DCs, which were cultured in an incubator supplemented with 5% CO_2_ at 37°C for 2 h. Following that, different concentrations of MSCs (MSC : DC ratios were 1 : 1, 1 : 10, 1 : 50, and 1 : 100, resp.) were added to the upper chamber. To determine the coculture time effect between MSCs and DCs, MSCs were added (MSCs : DCs were 1 : 10) on the 3rd and 5th days, respectively. DCs and culture supernatants were collected on the sixth day, and effect of MSCs on DCs was measured at different concentrations of MSCs and coculture times.

To explore whether MSCs exert immunoregulatory effect by gal-1, coculture was divided into four groups: (a) DC, (b) MSC + DC, (c) Gal-1 + DC, and (d) MSC + DC + Gal-1 inhibitor Thiodigalactoside (TDG). DCs were placed in lower chamber, and MSCs (MSC : DC 1 : 10) were added to upper chamber after 2 h, or recombinant Gal-1 protein was added directly to DC medium (final concentration of 3 *μ*M, Sigma, USA), or MSC + TDG (final concentration of 50 *μ*M, Sigma, USA). On the sixth day of coculture, DCs and culture supernatants were collected to observe whether MSCs played a role in DCs and to identify the possible mechanism.

### 2.5. Morphology of DCs Observed under an Electron Microscope

DCs were collected on the sixth day and resuspended in phosphate-buffered saline (PBS). The cell suspension was added to a coverslip coated with polylysine (Sigma, USA), placed in a 37°C electric constant-temperature oven for 15 min, gently washed three times with precooled PBS, and fixed with precooled 3% glutaraldehyde (pH 7.4) at 4°C for 1 h. The cells were fixed with 1% osmium acid for 1 h after rinsing, followed by stepwise dehydration, replacement, drying, and coating with different concentrations of ethanol. The morphological characteristics of the cells were observed under an electron microscope (Hitachi, Japan).

### 2.6. Flow Cytometry

Expression of CD90, CD105, CD45, and CD11b/c (eBioscience, USA) on MSCs surface was assessed by flow cytometry (Becton Dickinson, USA). The expression of CD11b/c, CD80, CD83, CD86, and MHC II (eBioscience, USA) on DCs surface was identified using flow cytometry. Each group of DCs were collected on the sixth day. After washing with PBS, the cells were immunolabeled with monoclonal anti-CD80, anti-CD83, anti-CD86, and anti-MHC II (eBioscience, USA) antibodies, as well as their isotype control antibodies. After that they were incubated in darkness at 4°C for 30 min and were detected using FACSCalibur flow cytometer.

### 2.7. Mixed Lymphocyte Reaction

The spleen was obtained from C57 mouse under sterile condition to prepare spleen single cell suspension. The cells were resuspended in RPMI 1640 medium with a cell density of 2 × 10^6^/mL. These cells were the responders and DCs cultured in different groups were stimulating cells. The cells cultured in the RPMI 1640 medium were adjusted to a density of 2 × 10^5^/mL, followed by the addition of 25 *μ*g/mL of Mitomycin (Sigma, USA), and then they were incubated at 37°C for 1 h. The ratio of stimulating cells to responders was 1 : 10. Meanwhile, empty well (with only RPM 1640 medium) and negative control group (with responders only) were set up. The cells were incubated at 37°C in a 5% CO_2_ incubator for 72 h. Then, 20 *μ*L of cell counting kit 8 (DOJINDO, Japan) was added to each well, and cells were incubated in a 5% CO_2_ incubator at 37°C for 4 h. The absorbance at 450 nm was measured using a microplate reader (Aware Inc., USA).

### 2.8. Enzyme-Linked Immunosorbent Assay

On the sixth day of coculture, the supernatants was collected, and IL-10, IL-12, and Gal-1 levels in coculture medium were measured according to kit instructions (R&D Co., USA). The data were read at OD_450_.

### 2.9. Immunofluorescence

During cell culture, sterilized coverslips coated with polylysine were placed at bottom of the culture plates. After 6 days, the coverslips were collected from different groups and washed with PBS, followed by incubation with 4% formaldehyde for 20 min. After this, the coverslips were washed with PBS and incubated with 10% FBS in PBS at room temperature for 1 h. They were incubated with the anti-Gal-1 primary antibody (1 : 200, Abcam, USA) at 4°C overnight. On the next day, they were washed with PBS, followed by addition of secondary antibody (1 : 500, p-phycoerythrin-labeled goat anti-rabbit immunoglobulin G, eBioscience, USA), and incubated at room temperature in darkness for 1 h. After washing with PBS, 100 uL of 4′,6-diamidino-2-phenylindole (DAPI) (10 *μ*g/mL, Sigma, USA) was added to each coverslip, followed by staining at room temperature for 15 min and observation under a fluorescence microscope (Nikon, Japan).

### 2.10. Real-Time Polymerase Chain Reaction

The DCs were collected from each group on sixth day of coculture, and the mRNA was extracted by a one-step method using Trizol (Invitrogen, USA) reagent, followed by reverse transcription in a 10-*μ*L reaction system according to instructions of the PrimeScript Real-Time Reagent Kit (Takara Co., Ltd., Japan). The Gal-1 of standard sample and the sample to be tested were polymerase chain reaction- (PCR-) amplified on an ABI 7500 Real-Time PCR instrument (ABI, USA) under the same reaction condition using glyceraldehyde-3-phosphate dehydrogenase (GAPDH) as the internal reference gene; 20 *μ*L reaction system (SYBR Premix Ex Taq FQ-PCR Kit, Takara Co., Ltd.) was used. The gene sequences were as follows: Gal-1: forward, TGAACCTGGGAAAAGACAGC, and reverse, TAGTGGAAACTGGTCCGACT; GAPDH: forward, TGGTGAAGGTCGGTGTGAAC, and reverse, GTGAGTGGAGTCATACTGGAAC.

### 2.11. Western Blotting

DCs were collected from each group, and protein lysate (Pik Wan Biotechnology Research Institute, China) was added after washing with precooled PBS. Each sample was quantified using the Protein Assay Kit (Pik Wan Biotechnology Research Institute). The proteins extracted from DCs were gel-electrophoresed with 15% polyacrylamide (sodium dodecyl sulfate polyacrylamide gel electrophoresis), wet-transferred to polyvinylidene difluoride (PVDF) membranes (Millipore, USA), treated with Tris-buffered saline with Tween 20 (TBST) solution containing 5% nonfat dry milk (Bio-Rad, USA), and blocked for 2 h at room temperature. After blocking, some amount of mouse Gal-1 antibody (1 : 1000, Abcam, USA) and antibodies to p38 MAPK (1 : 1000), Phospho-p38 MAPK (1 : 1000), extracellular signal-regulated kinase (ERK)1/2 (1 : 1000), Phospho-ERK1/2 (1 : 1000), c-Jun N-terminal kinase (JNK) (1 : 1000), and Phospho-ERK1/2 (1 : 1000) (Cell Signaling Technology, USA) diluted in TBST were added and incubated at 4°C overnight. On the next day, the membranes were washed with TBST and incubated with horseradish peroxidase–labeled goat anti-rabbit secondary antibody (1 : 5000, Abcam, USA) for 2 h at room temperature. After washing, enhanced chemiluminescence (Millipore, USA) was used for development. The PVDF membrane was imaged using the Bio-Rad automated gel imaging system GelDoxXR+ (Bio-Rad, USA), and the results were analyzed using Image Lab Software version 5.0 (Bio-Rad). The gray scale values of the bands were measured. The relative expression of proteins was defined as gray scale value of target protein/gray scale value of GAPDH, or gray scale value of target protein/gray scale value of internal reference.

### 2.12. Statistical Analysis

Statistical analysis was performed using GraphPad Prism Software (CA, USA). All data were presented as mean ± standard deviation (*x* ± SD). One-way ANOVA was used to compare the differences between groups. Dunnett-*t* was used for pairwise comparisons. A *P* value < 0.05 was considered statistically significant (*∗* or # indicated *P* < 0.05, and *∗∗* or ## indicated *P* < 0.01). Each in vitro coculture group had at least three to four independent coculture systems.

## 3. Results

### 3.1. MSCs Were Identified by Morphology and Flow Cytometry

**Figure 1:**
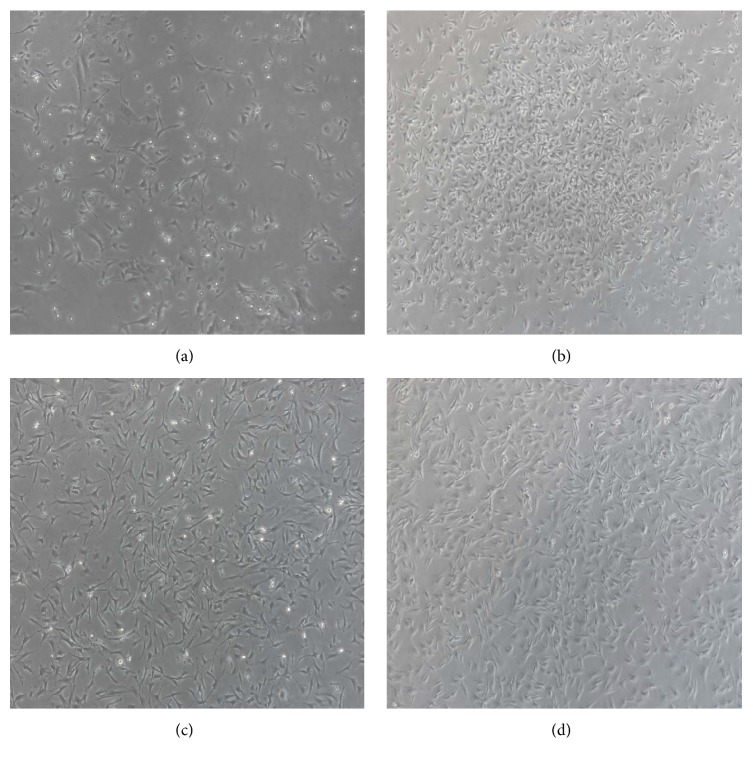
*MSCs were cultured by the whole bone marrow adherent method (50x)*. (a) MSCs cultured for 48 h. (b) MSCs cultured till the seventh day: the cells increased significantly and formed colonies. (c) MSCs cultured till the 11th day: the cells exhibited a swirling or radial arrangement. (d) MSCs passed to the third generation and cultured till the seventh day.

**Figure 2:**
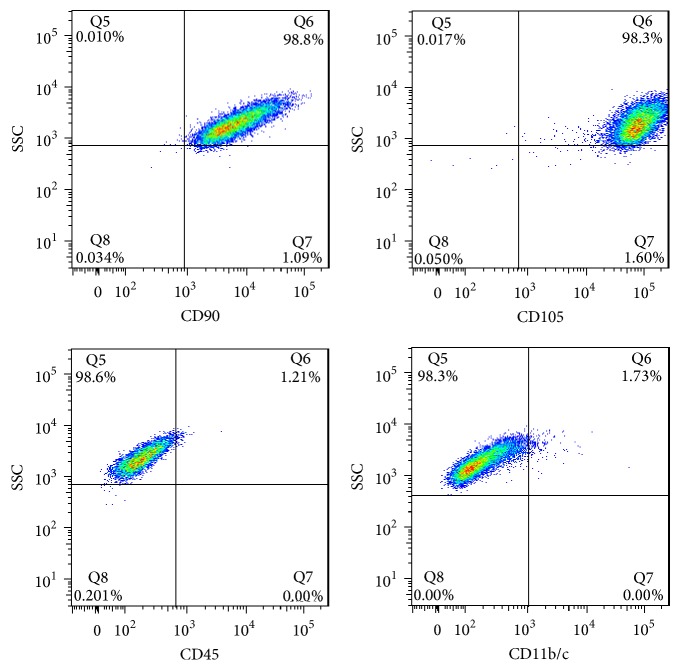
*CD phenotype of mouse MSCs detected by flow cytometry*. The results showed that MSCs expressed CD90 and CD105 but did not express CD45 and CD11b/c.

The adherent cells obtained from bone marrow of BALB/c mice under standard culture conditions became long fusiform on seventh day of culture. The cells formed obvious colonies, with evident cell division and proliferation. These cells had abundant cytoplasm and big and oval nuclei (Figure 1(b)). From 10th to 12th day, the cells covered 80%–90% of dish bottom and fused in a swirling or radial arrangement (Figure 1(c)). The third generation of MSCs was examined using flow cytometry (Figure 1(d)), and as shown in Figure 2, MSCs expressed CD90 and CD105 (the ratio of CD90 and CD105 was 98.8% and 98.3%, resp.) but did not express CD45 and CD11b/c (the ratio of CD45 and CD11b/c positive cells was 1.21% and 1.73%, resp.). These results indicated that the isolated and cultured MSCs had a typical expression profile of MSCs.

### 3.2. DCs Were Identified by Morphology and Flow Cytometry

**Figure 3:**
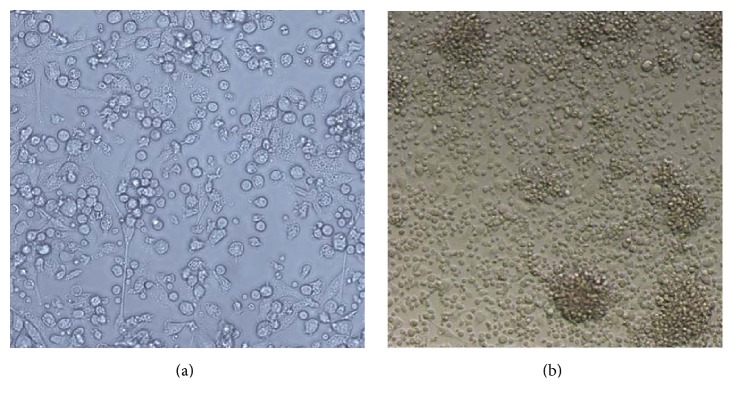
*Isolating and culturing of DCs from the bone marrow*. (a) DCs cultured for 24 h (100x). (b) MSCs cultured till the seventh day: the cells actively proliferated, grew by loosely adhering to the walls, clustered, and existed as small colonies (50x).

**Figure 4:**
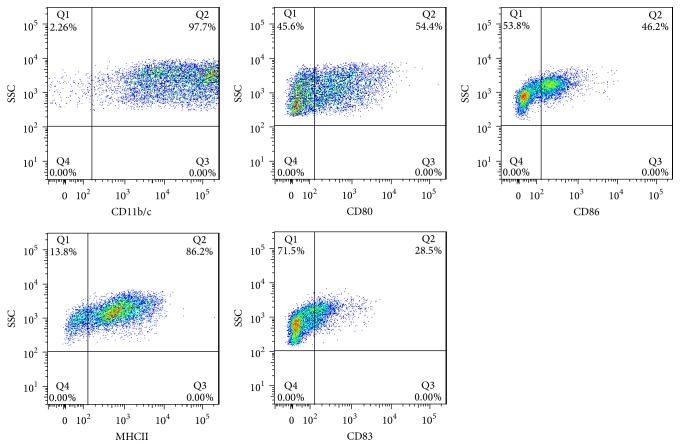
*CD phenotype of mouse DCs detected by flow cytometry*. The results showed that CD11b/c was expressed in a high percentage of DCs, MHC II was expressed in a moderate percentage of DCs, and CD80, CD83, and CD86 were expressed in a low percentage of DCs.

DCs were isolated from bone marrow of the BALB/c mouse tibia and fibula and cultured for 5 days. Most of the cells were round under a microscope, and some of them showed a burr-like protuberance on the edge of the cells. As shown in Figure 3, the cells actively proliferated, grew by loosely adhering to the walls, clustered, and existed as small colonies. The flow cytometry results showed that the positive rate of CD11b/c, CD80, CD83, CD86, and MHC II in DCs was 97.7%, 54.4%, 28.5%, 46.2%, and 86.2%, respectively (Figure 4). These results indicated that the isolated and cultured DCs had a typical expression profile of DCs, providing a basis for the following functional experiments.

### 3.3. MSCs Downregulate the Expression of Costimulatory Molecules on the Surface of DCs

**Figure 5:**
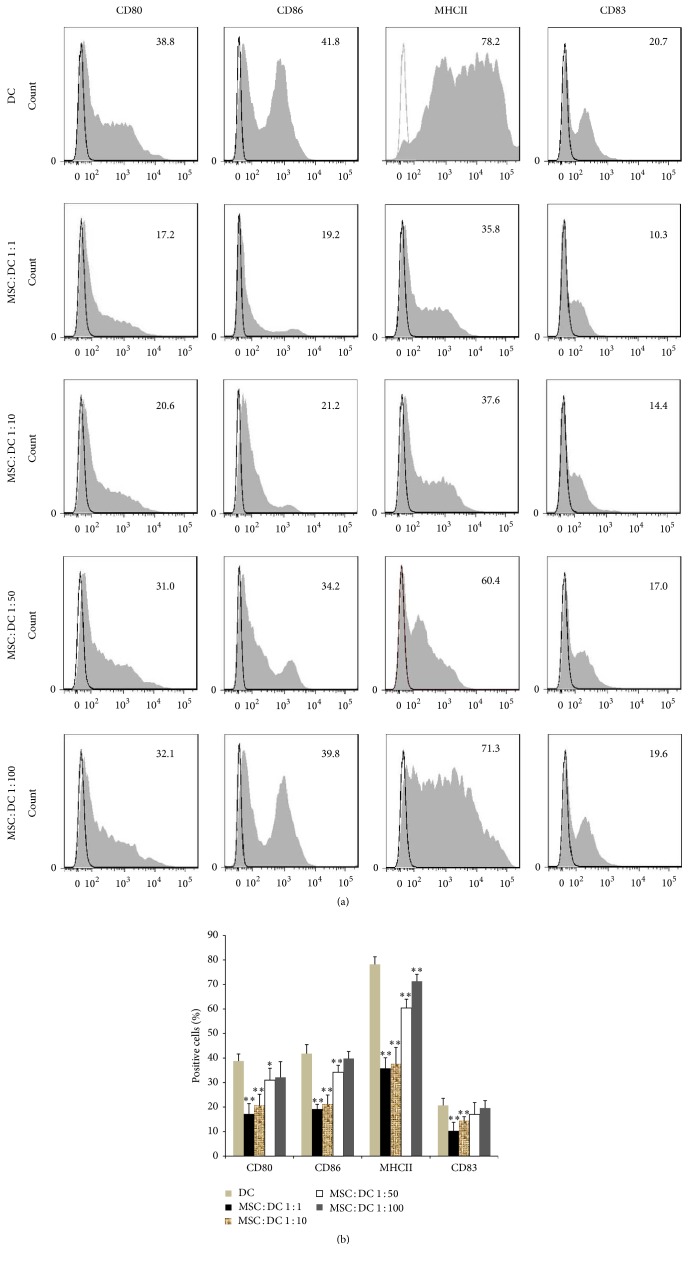
*Effect of different MSC and DC coculture concentrations on DC phenotype was detected by flow cytometry*. (a, b) The expression of various biomarkers on the surface of DCs was decreased with the increase in the ratio of MSCs to DCs in the coculture system (*n* = 5, ^*∗*^*P* < 0.05 versus DC only group, ^*∗∗*^*P* < 0.01 versus DC only group).

As shown in Figure 5, when MSCs were added for coculture 2 h after DCs were plated (with the MSC : DC ratios of 1 : 1 and 1 : 10), the expression of CD80, CD83, CD86, and MHC II on the surface of DCs was significantly lower than that in DCs of DC only group detected by flow cytometry. At the MSC : DC ratio of 1 : 50, the expression of CD80, CD86, and MHC II was significantly lower than that in DCs of DC only group, whereas the expression of CD83 was not significantly different. At the MSC : DC ratio of 1 : 100, although the expression of MHC II was decreased compared with that in DC only group, the expression of other markers showed no significant difference. This result suggested that the expression of various biomarkers on the surface of DCs was decreased with the increase in ratio of MSCs to DCs in the coculture system. However, the expression of CD83 at the MSC : DC ratio of 1 : 1 was increased compared with that at the MSC : DC ratio of 1 : 10 (*P* < 0.05); the expression of other biomarkers showed no significant difference. This indicated that when the concentration of MSCs increased to a certain level (1 : 10), the effect of MSCs on expression of costimulatory molecules no longer increased with the increase of MSCs to DCs ratio.

**Figure 6:**
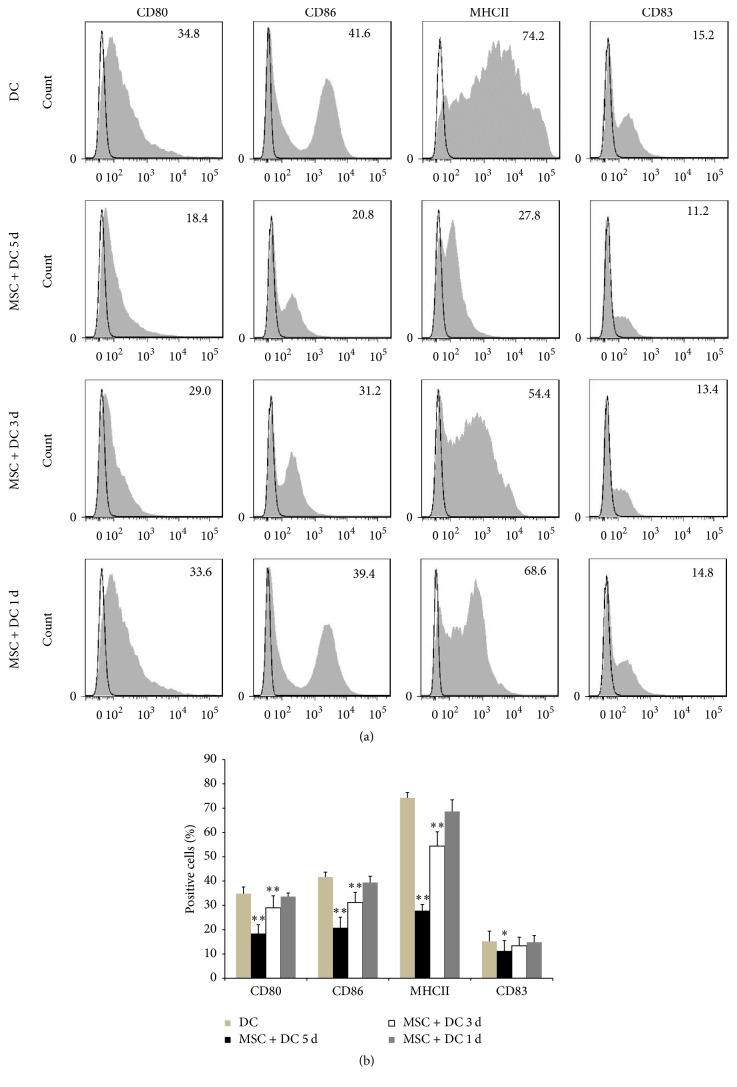
*Effect of MSC and DC coculture time on DC phenotype was detected by flow cytometry*. (a, b) MSC + DC 5 d, MSC + DC 3 d, and MSC + DC 1 d refer to MSCs and DCs coculture for 5 days, 3 days, and 1 day, respectively. The effect of MSCs on the costimulatory molecules on the surface of DCs was more significant as the time of MSC and DC coculture increased. (*n* = 5, ^*∗*^*P* < 0.05 versus DC only group, ^*∗∗*^*P* < 0.01 versus DC only group).

Following above, the ratio of MSC : DC was set at 1 : 10, and MSCs were added to bone marrow-derived DCs at 2 h, 3 days, and 5 days after isolation for coculture. As shown in Figure 6, when MSCs were added 2 h after DCs were plated, the expression of CD80, CD83, CD86, and MHC II was significantly lower than that of DCs in DC only group. When MSCs were added on the third day for coculture, the expression of CD80, CD86, and MHC II on the surface of DCs was significantly lower than that of DCs in DC only group, whereas the expression of CD83 was not significantly different. When MSCs were added on the fifth day for coculture, the expression of CD80, CD86, MHC II, and CD83 on the surface of DCs showed no significant difference compared with that in DC only group. These results suggested that effect of MSCs on the costimulatory molecules on the surface of DCs was more significant as the time of MSC and DC coculture increased.

### 3.4. MSCs Increase the Secretion of IL-10 and IL-12 and the Level of Gal-1 in DC Culture Supernatants

**Figure 7:**
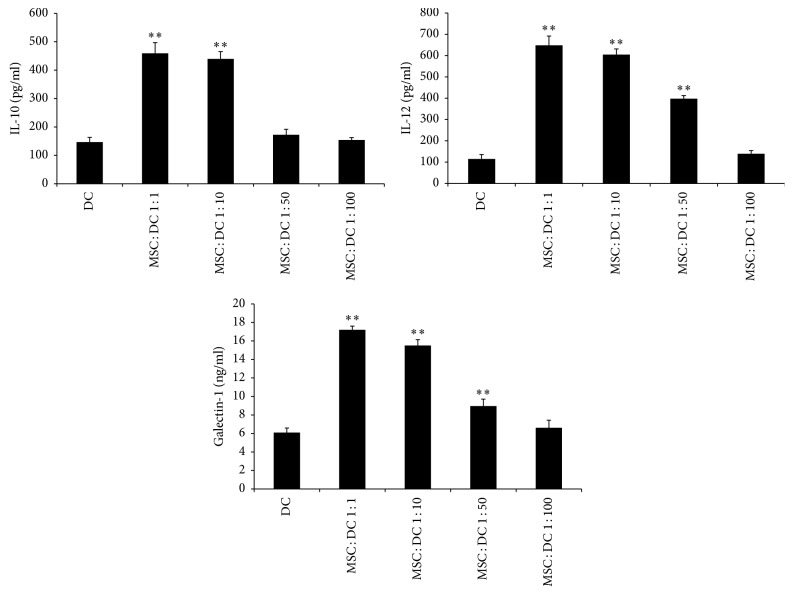
*Concentrations of IL-10, IL-12, and Gal-1 in cell culture supernatants (mean ± SD)*. With the increase in the ratio of MSCs to DCs in the coculture system, the levels of IL-10, IL-12, and Gal-1 also increased (*n* = 5, ^*∗∗*^*P* < 0.01 versus DC only group).

MSCs were cocultured with DCs in vitro using Transwell chambers. As shown in Figure 7, when MSCs were cocultured with DCs 2 h after DCs were plated, at the MSC : DC ratio of 1 : 100, the levels of IL-10, IL-12, and Gal-1 (153.84 ± 8.62, 138.96 ± 15.39, and 6.62 ± 0.81, resp.) in the supernatants of DCs were not significantly different from those in DC only group (146.58 ± 16.51, 114.63 ± 21.15, and 6.09 ± 0.48, resp.). At the MSC : DC ratio of 1 : 50, the levels of IL-12 and Gal-1 (397.65 ± 14.75 and 8.95 ± 0.75, resp.) in the supernatants of DCs were increased compared with those in DC only group, whereas the level of IL-10 (172.30 ± 19.53) was not significantly different. At the MSC : DC ratio of 1 : 10 and 1 : 1, the levels of IL-10, IL-12, and Gal-1 (MSC : DC 1 : 10 439.27 ± 25.63, 604.86 ± 26.45, 15.49 ± 0.64, resp.; MSC : DC 1 : 1 459.08 ± 37.91, 648.83 ± 43.29, 17.2 ± 0.38, resp.) in the supernatants of DCs were significantly increased compared with those in DC only group. The level of Gal-1 was higher at the MSC : DC ratio of 1 : 1 than at the MSC : DC ratio of 1 : 10, whereas the levels of IL-10 and IL-12 showed no significant difference between the two groups. This suggested that, with the increase in the ratio of MSCs to DCs in the coculture system, the levels of IL-10, IL-12, and Gal-1 were also increased; but when the concentration of MSCs reached a certain value (MSC : DC ratio of 1 : 10), the levels of IL-10 and IL-12 did not increase with the increase in the ratio of MSCs to DCs. Therefore, the MSC : DC ratio of 1 : 10 was used as the concentration ratio for subsequent experiments.

**Figure 8:**
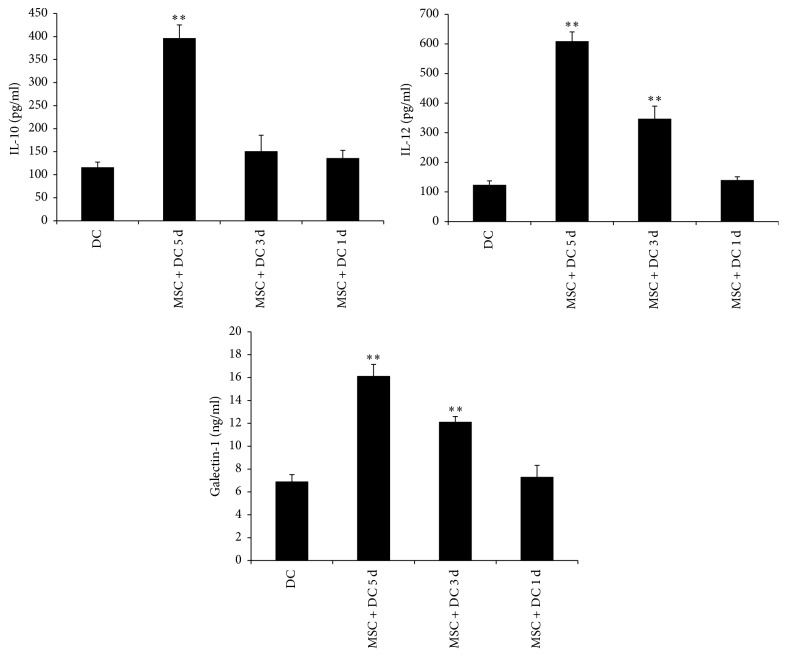
*Concentrations of IL-10, IL-12, and Gal-1 in cell culture supernatants were determined via ELISA*. MSC + DC 5 d, MSC + DC 3 d, and MSC + DC 1 d refer to MSCs and DCs coculture for 5 days, 3 days, and 1 day, respectively. At the same concentration and under the same conditions when the MSC and DC coculture time was longer, the increase in the IL-10, IL-12, and Gal-1 levels of the coculture system was more significant (mean ± SD) (*n* = 5, ^*∗∗*^*P* < 0.01 versus DC only group).

In the following experiments the MSC : DC ratio was set at 1 : 10, and MSCs were added to DCs for coculture at 2 h, 3 days, and 5 days after DCs were plated. As shown in Figure 8, when MSCs were added on the fifth day, the levels of IL-10, IL-12, and Gal-1 (136.02 ± 16.89, 140.18 ± 11.19, and 7.32 ± 0.99, resp.) in the DC supernatants did not change significantly compared with those in DC only group (116.17 ± 11.21, 123.74 ± 13.61, and 6.91 ± 0.59, resp.). When MSCs were added for coculture on the third day, the IL-12 and Gal-1 levels (347.08 ± 42.79, 12.13 ± 0.46, resp.) in the DC supernatants were significantly higher than those in DC only group, whereas the IL-10 level (150.96 ± 34.74) was not significantly different. When MSCs were added for coculture 2 h after isolation, the levels of IL-10, IL-12, and Gal-1 (396.49 ± 28.83, 608.98 ± 31.74, and 16.13 ± 1.01, resp.) in the DC supernatants were significantly increased compared with those in DC only group. This suggested that, at the same concentration and under the same condition, when the MSC and DC coculture time was longer, the increase in the IL-10, IL-12, and Gal-1 levels of the coculture system was more significant.

### 3.5. MSCs Reduce the Proliferative Effect of DCs on T Cells

**Figure 9:**
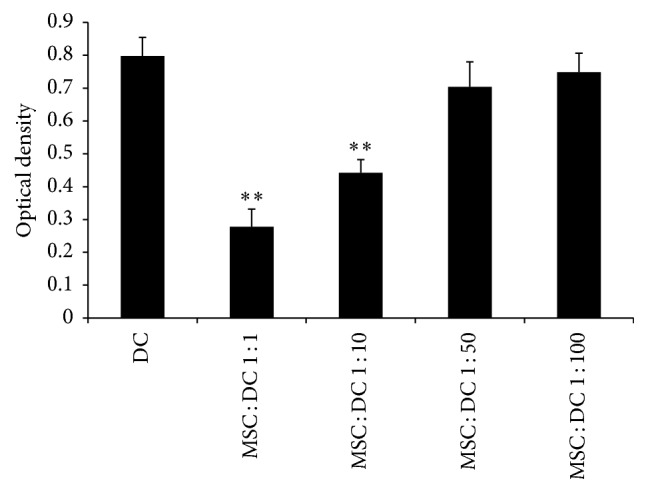
*Mixed lymphocyte reaction assay was used to examine the DC-stimulated T cell proliferation response*. With the increase in the ratio of MSCs to DCs, the proliferative effect of DCs on T cells was significantly reduced (*n* = 5, ^*∗∗*^*P* < 0.01 versus DC only group).

The one-way mixed lymphocyte culture/reaction (MLC/MLR) is an in vitro method to examine the T cell stimulatory capacity of DC in the coculture system by assaying T cell proliferation. As shown in Figure 9, when the MSC : DC ratio was 1 : 100 and 1 : 50, the OD values after DCs stimulated T cells (0.748 ± 0.058, 0.704 ± 0.076) were not significantly different compared with those in DC only group (0.798 ± 0.056). When the MSC : DC ratio was 1 : 10 and 1 : 1, the OD values after DCs stimulated T cells (0.442 ± 0.04 and 0.278 ± 0.054, resp.) were significantly lower than those in DC only group (*P* < 0.05). This suggested that, with the increase in the ratio of MSCs to DCs, the proliferative effect of DCs on T cells was significantly reduced.

**Figure 10:**
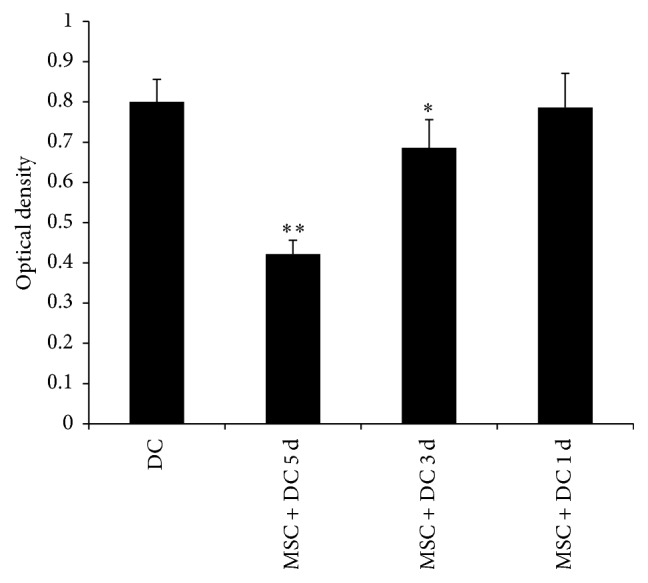
*Mixed lymphocyte reaction assay was used to examine the DC-stimulated T cell proliferation response*. MSC + DC 5 d, MSC + DC 3 d, and MSC + DC 1 d refer to MSCs and DCs coculture for 5 days, 3 days, and 1 day, respectively. As the MSC and DC coculture time increased, the proliferative effect of DCs on T cells was significantly reduced (*n* = 5, ^*∗∗*^*P* < 0.01 versus DC only group, ^*∗*^*P* < 0.05 versus DC only group).

Next, the MSC : DC ratio was set as 1 : 10, and MSCs were added for coculture at 2 h, 3 days, and 5 days. As shown in Figure 10, when MSCs were added at day 5, the OD value after DCs stimulated T cells was 0.786 ± 0.085, which was not significantly different from that of DC only group (0.8 ± 0.056). When MSCs were added 2 h and 3 days after DCs were plated, the OD values after DCs stimulated T cells were 0.422 ± 0.034 and 0.686 ± 0.069, respectively, which were lower than those of DC only group. This suggested that the inhibitory effect on the DC function was more obvious as the MSC and DC coculture time increased.

The present study found that, after coculturing MSCs with DCs, the expression of CD80, CD86, and MHCII on the surface of DCs decreased, and the effect of DCs on T cell proliferation was also weakened, suggesting MSCs could inhibit the maturation and function of DCs. Based on these results, the MSC : DC ratio in the following experiments was set as 1 : 10, and MSCs were added for coculture at 2 h after DCs were plated.

### 3.6. Gal-1 Downregulates the Expression of Costimulatory Molecules on the Surface of DCs

**Figure 11:**
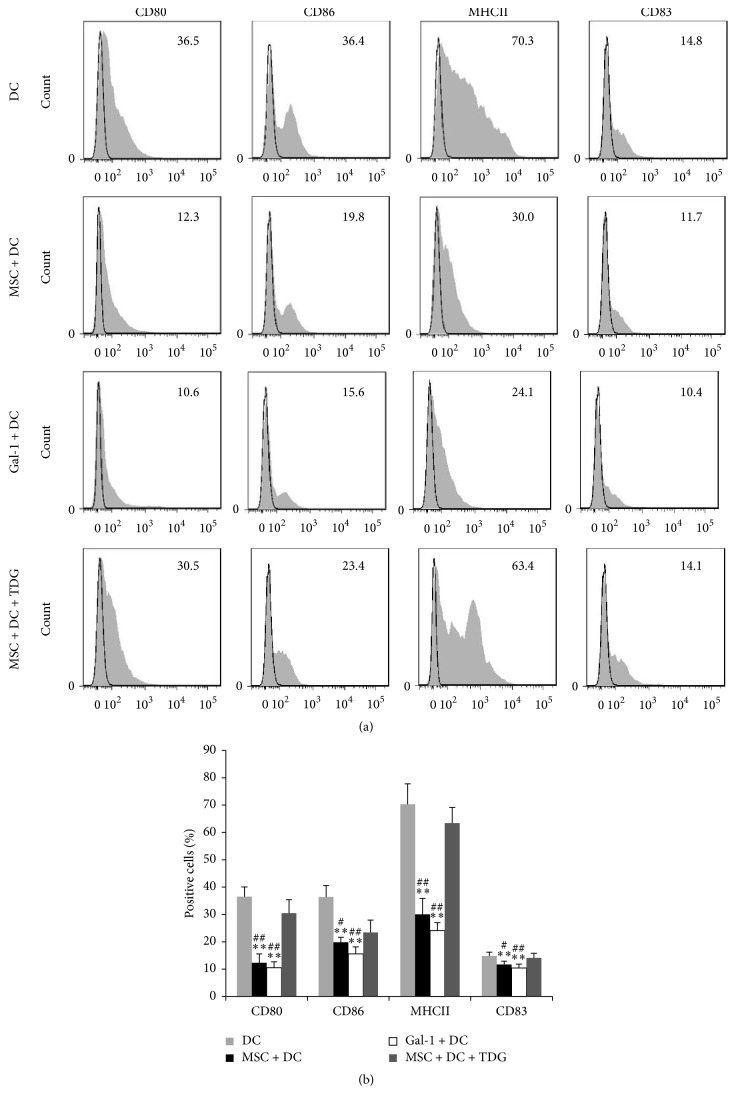
*Effect of Gal-1 on DC phenotype in each group was detected by flow cytometry*. (a, b) MSCs and Gal-1 downregulate the expression of costimulatory molecules on the surface of DCs. After adding TDG, the inhibitory effect of MSCs on the costimulatory molecules was compromised (*n* = 5, ^*∗∗*^*P* < 0.01 versus DC only group, ^##^*P* < 0.01 versus MSC + DC + TDG group, ^#^*P* < 0.05 versus MSC + DC + TDG group).

As shown in Figure 11, the expression of CD80, CD83, CD86, and MHC II on the surface of DCs in the MSC + DC and Gal-1 + DC groups was significantly lower than that in DC only group. When Gal-1 inhibitor TDG was added to the MSC + DC group, the expression of costimulatory molecules on the surface of DCs was more significantly increased than that of the MSC + DC and Gal-1 + DC groups. These results showed that both Gal-1 and MSCs inhibited the expression of costimulatory molecules on the surface of DCs. After adding the Gal-1 inhibitor, the inhibitory effect of MSCs on the costimulatory molecules was compromised, suggesting that MSCs might play inhibitory role through Gal-1.

### 3.7. Gal-1 Increases the Level of IL-10 and IL-12 in DC Culture Supernatants

**Figure 12:**
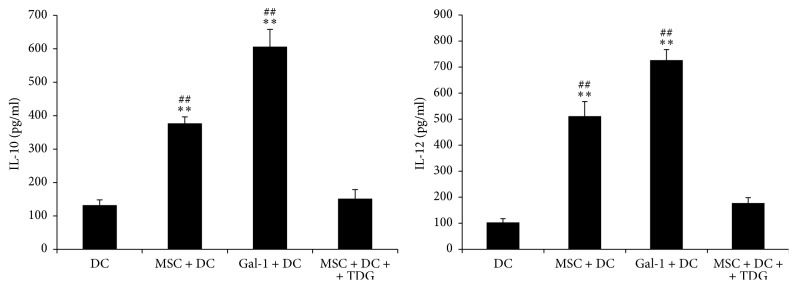
*Concentrations of IL-10 and IL-12 in cell culture supernatants were determined via ELISA*. The levels of IL-10 and IL-12 in the supernatants of the MSC + DC and Gal-1 + DC groups significantly increased compared with those in DC only group. After adding TDG, the levels of IL-10 and IL-12 in the supernatant of the MSC + DC + TDG group were significantly lower than those of the MSC + DC and Gal-1 + DC groups (mean ± SD) (*n* = 5, ^*∗∗*^*P* < 0.01 versus DC only group, ^##^*P* < 0.01 versus MSC + DC + TDG group.

As shown in Figure 12, the levels of IL-10 and IL-12 in the supernatants of the MSC + DC (376.96 ± 19.57 and 511.58 ± 56.27, resp.) and Gal-1 + DC groups (606.13 ± 51.56 and 726.89 ± 40.61, resp.) were significantly increased compared with those in DC only group (132.47 ± 15.96 and 103.63 ± 14.60, resp.). After adding TDG, the levels of IL-10 and IL-12 in the supernatants of the MSC + DC + TDG group (151.68 ± 27.06 and 177.61 ± 21.04, resp.) were significantly lower than those of the MSC + DC and Gal-1 + DC groups, suggesting that Gal-1 promoted the secretion of IL-10 and IL-12 from DCs.

### 3.8. Gal-1 Reduces the Proliferative Activity of T Cells Stimulated by DCs

**Figure 13:**
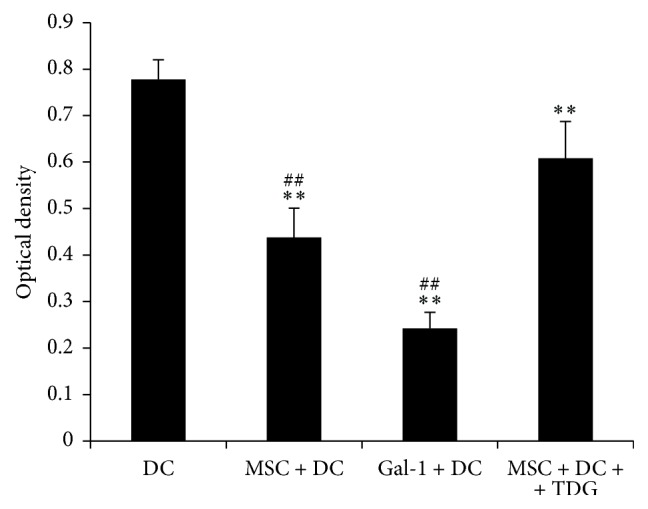
*Mixed lymphocyte reaction assay detected T cell proliferation response stimulated by DCs*. The DCs of the MSC + DC and Gal-1 + DC groups only slightly stimulated the proliferation of allogeneic T cells. After adding TDG, the DCs in the MSC + DC + TDG group stimulated the fast proliferation of T cells (*n* = 5, ^*∗∗*^*P* < 0.01 versus DC only group, ^##^*P* < 0.01 versus MSC + DC + TDG group).

As shown in Figure 13, the mixed lymphocyte reaction assay results showed that the DCs of the MSC + DC (OD value 0.438 ± 0.063) and Gal-1 + DC groups (OD value 0.242 ± 0.035) only slightly stimulated the proliferation of allogeneic T cells; in these two groups, the proliferation of allogeneic T cells stimulated by DCs was significantly lower than that in DC only group (OD value 0.778 ± 0.042). After adding TDG, the DCs in the MSC + DC + TDG group (OD value 0.608 ± 0.079) stimulated the fast proliferation of T cells, which was significantly different compared with those in the MSC + DC and Gal-1 + DC groups, suggesting that MSCs inhibited the function of DCs by secreting Gal-1.

### 3.9. MSCs Increase the Expression of Gal-1 in DCs

**Figure 14:**
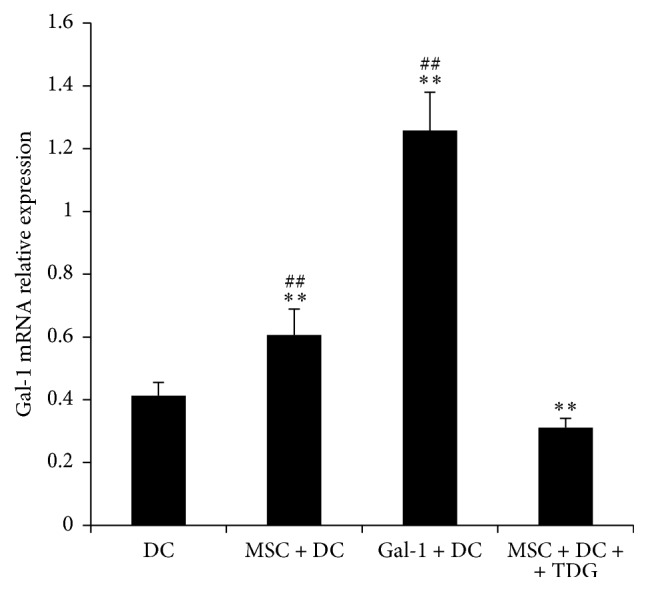
*Expression of Gal-1 mRNA in DCs of each experimental group was determined via real-time PCR*. The expression of Gal-1 mRNA was enhanced when MSCs or Gal-1 was cocultured with DCs but inhibited by TDG (*n* = 5, ^*∗∗*^*P* < 0.01 versus DC only group, ^##^*P* < 0.01 versus MSC + DC + TDG group).

As shown in Figure 14, the expression of Gal-1 mRNA in the MSC + DC group (0.606 ± 0.05) was significantly higher than that in DC control group (0.413 ± 0.042); the expression of Gal-1 mRNA in the Gal-1 + DC group (1.257 ± 0.122) was significantly higher than that in DC only group. After adding TDG, the expression of Gal-1 mRNA in MSC + DC + TDG group (0.311 ± 0.029) was significantly decreased compared with that in DCs, MSC + DC, and Gal-1 + DC groups.

**Figure 15:**
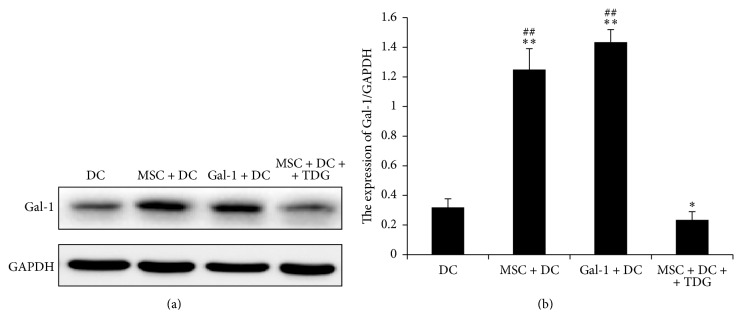
*Expression of Gal-1 protein in each group of DCs was determined via Western Blot*. (a) Western blot detected the expression of Gal-1 protein in DCs of each group, with the expression of GAPDH as the internal reference. (b) Relative expression was obtained by dividing the gray scale value of each band by the GAPDH value (*n* = 5, ^*∗∗*^*P* < 0.01 versus DC only group, ^*∗*^*P* < 0.05 versus DC only group, ^##^*P* < 0.01 versus MSC + DC + TDG group).

As shown in Figure 15, expression of Gal-1 protein on DCs in the MSC + DC and Gal-1 + DC groups (1.248 ± 0.141 and 1.433 ± 0.085, resp.) was significantly higher than that in DC only group (0.319 ± 0.057). After adding TDG, the protein expression of Gal-1 in the MSC + DC + TDG group (0.235 ± 0.054) was significantly lower than that in the MSC + DC, Gal-1 + DC (*P* < 0.05), and control groups.

The results of RT-PCR and Western blot showed that the expression of Gal-1 mRNA and protein on DCs was enhanced when MSCs were cocultured with DCs but inhibited by the Gal-1 inhibitor TDG.

### 3.10. Immunofluorescence Detected Differential Expression of Gal-1 on the Surface of DCs

**Figure 16:**
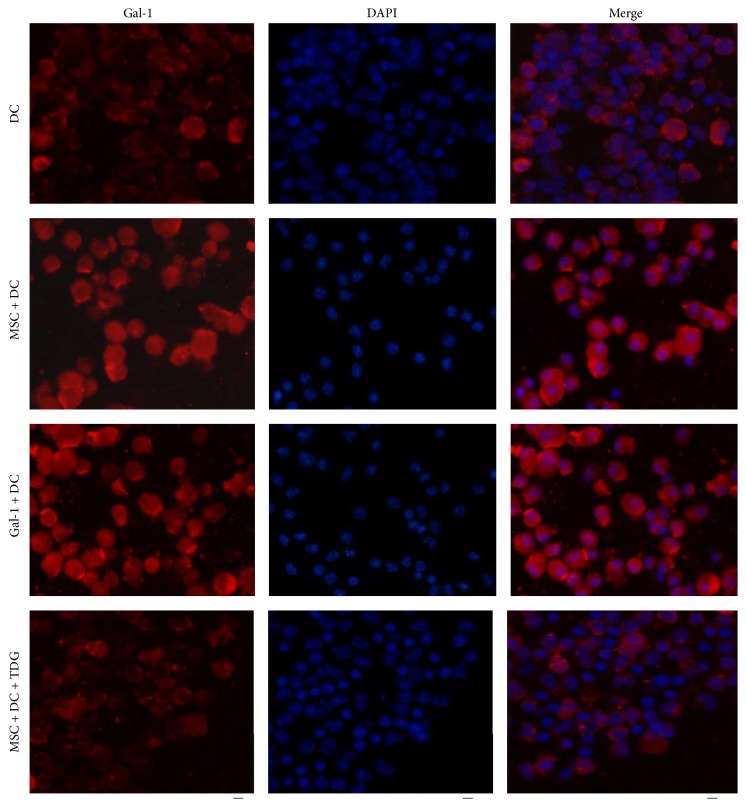
*Localized expression of Gal-1 in DCs of each experimental group was determined via immunofluorescence*. The expression of Gal-1 protein on the surface of DCs was enhanced when MSCs or Gal-1 was cocultured with DCs but inhibited by TDG.

As shown in Figure 16, immunofluorescence double staining and DAPI staining were performed in DC control, MSC + DC, Gal-1 + DC, and MSC + DC + TDG groups. The results showed that expression of Gal-1 protein on the surface of DCs of MSC + DC and Gal-1 + DC groups was higher than that of DC group. The increase in the expression of Gal-1 protein was more significant especially in the Gal-1 + DC group. After adding TDG, the expression of Gal-1 protein on the surface of DCs of the MSC + DC + TDG group was significantly lower than that of MSC + DC, Gal-1 + DC, and DC only group.

### 3.11. Gal-1 Activated the ERK Pathway and Inhibited the p38 MAPK Pathway in DCs

**Figure 17:**
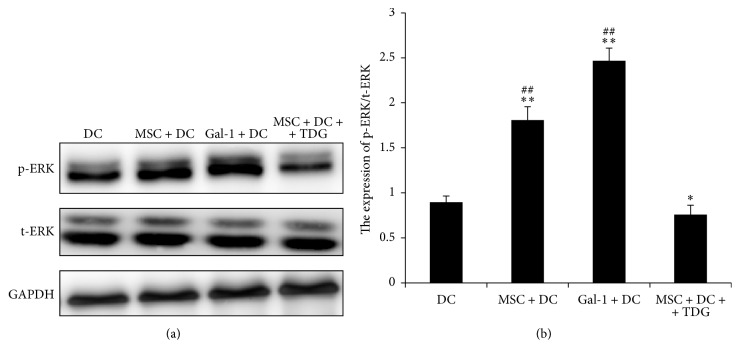
*Expression of ERK phosphorylation in each group of DCs was determined via Western Blot*. (a) Expression of ERK phosphorylation in DCs was detected by Western blot, with the expression of t-ERK as a reference. (b) Relative expression was obtained by dividing the gray scale value of each band by the t-ERK level (*n* = 5, ^*∗∗*^*P* < 0.01 versus DC only group, ^*∗*^*P* < 0.05 versus DC only group, ^##^*P* < 0.01 versus MSC + DC + TDG group).

The MAPK signaling pathway, including ERK1/2, p38 MAPK, and JNK, is known to play an important role in differentiation of DCs and regulation of cytokine secretion [16]. Therefore, this study investigated whether Gal-1 played its regulatory role through the MAPK pathway. The expression of ERK, p38 MAPK, and JNK was examined in each group. As shown in Figure 17, the level of phosphorylated ERK1/2 in the MSC + DC and Gal-1 + DC groups (1.807 ± 0.151 and 2.466 ± 0.143, resp.) was significantly higher than that in DC only group (0.897 ± 0.067). Specially speaking, the increase in ERK1/2 phosphorylation in the Gal-1 + DC group was more significant, suggesting that Gal-1 activated the ERK pathway. After adding TDG, the level of ERK phosphorylation in DCs of the MSC + DC + TDG group (0.759 ± 0.104) was significantly lower than that in the MSC + DC, Gal-1 + DC (*P* < 0.01), and DC only group.

**Figure 18:**
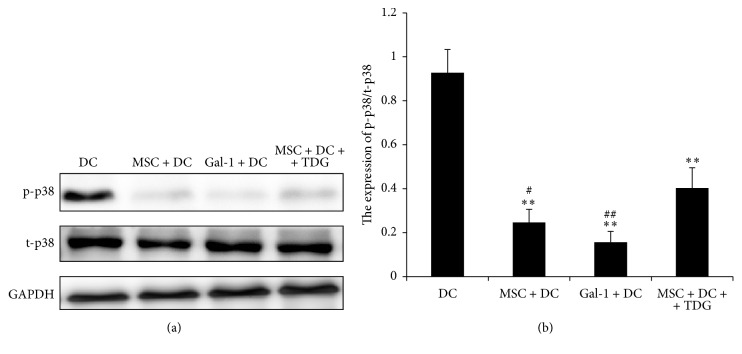
*Expression of P38 MAPK phosphorylation in each group of DCs was determined via Western Blot*. (a) Expression of P38 MAPK phosphorylation in DCs was detected by Western blot, with the expression of t-P38 MAPK as a reference. (b) Relative expression was obtained by dividing the gray scale value of each band by the t-P38 MAPK level (*n* = 5, ^*∗∗*^*P* < 0.01 versus DC only group, ^##^*P* < 0.01 versus MSC + DC + TDG group, ^#^*P* < 0.05 versus MSC + DC + TDG group).

As shown in Figure 18, the P38 MAPK phosphorylation level of DCs in the MSC + DC and Gal-1 + DC groups (0.246 ± 0.059 and 0.156 ± 0.049, resp.) was significantly lower than that in DC only group (0.928 ± 0.105), indicating that Gal-1 inhibited the p38 MAPK pathway. After adding TDG, the level of MAPK phosphorylation (0.403 ± 0.092) was significantly increased in the MSC + DC and Gal-1 + DC groups (*P* < 0.05, *P* < 0.01) but was still significantly lower compared with that in DC only group.

**Figure 19:**
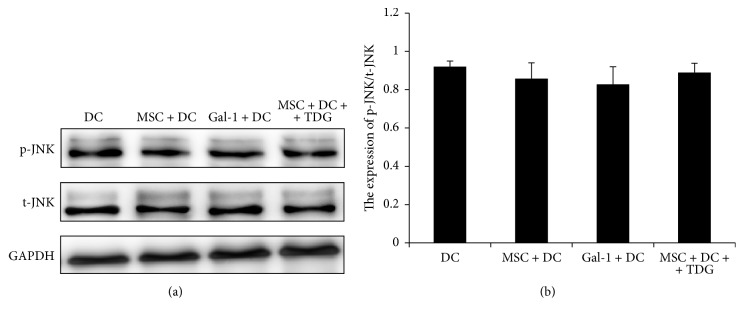
*Expression of JNK phosphorylation in each group of DCs was determined via Western Blot*. (a) Expression of JNK phosphorylation in DCs was detected by Western blot, with t-JNK as a reference. (b) Relative expression was obtained by dividing gray scale values of each band by the t-JNK level.

As shown in Figure 19, JNK phosphorylation levels in the MSC + DC and Gal-1 + DC groups (0.858 ± 0.081 and 0.828 ± 0.092, resp.) were not significantly different from those in DC only group (0.920 ± 0.029). After adding TDG, the level of JNK phosphorylation (0.889 ± 0.049) showed no significant difference compared with that in the MSC + DC, Gal-1 + DC, and DC only group.

## 4. Discussion

In this current study, MSCs and DCs were successfully isolated from mouse bone marrow, and MSCs via an in vitro coculture were found to inhibit the maturation and function of DCs through Gal-1 secretion by regulating the MAPK pathway in DCs.

Bone marrow MSCs are pluripotent stem cells derived from early developmental mesoderm. They have the ability of multidirectional differentiation and self-renewal. They also have low immunogenicity and a wide range of immunomodulatory effects and are therefore used for treating graft-versus-host diseases [17], tumors [18], autoimmune diseases such as rheumatoid arthritis [19], and systemic lupus erythematosus [20]. Studies found that the immunomodulatory effect of MSCs was mainly due to their ability to inhibit DC cells, B cells, natural killer cells, T cells, and other immune cells [21]. DCs are known to be the most powerful full-time antigen-presenting cells in vivo. Mature DCs have a strong immune activity: they can activate the initial T cells, induce T lymphocyte proliferation, start the immune response, and also promote the infiltration of inflammatory cells and the secretion of cytokines and influencing factors. Many studies [22, 23] indicated that DCs played an important role in immune response.

Previous studies showed that coculture of MSCs and DCs inhibited the differentiation, maturation, and activation of DCs by downregulating expression of costimulatory molecules such as CD80, CD86, and MHC II on the surface of DCs [24, 25]. The present study also found that after coculturing of MSCs with DCs, the expression of CD80, CD86, and MHCII on the surface of DCs was decreased with the increase in ratio of MSCs to DCs, and the effect of DCs on T cell proliferation was also weakened after coculturing with MSCs, suggesting that immunosuppressive effect of DCs was concentration dependent. Meanwhile, this study found that, at the same concentration ratio, the inhibitory effect on DC function was more obvious as the MSC and DC coculture times increased.

This present study demonstrated that MSCs inhibited the proliferative effects of DCs on T cells, but the exact mechanism was unclear. Previous studies showed that MSCs exerted an inhibitory effect through direct contact with DCs. Other studies reported that the effect of MSCs on DCs might be achieved through the secretion of some soluble factors. Kim et al. [26] found that MSCs secreted IL-10, which inhibited DC maturation and thereby suppressed T cell proliferation. Some studies also found that MSCs induced the immune tolerance of DCs by secreting prostaglandin E2, IL-6, and TGF-*β*. However, the role of Gal-1 in MSCs and DCs has been rarely explored.

Galectin is a member of the animal lectin family. It is involved in cell adhesion, growth regulation, immune response, and other biological processes [27]. Gal-1 is the first discovered member of the Galectin family, and its expression can be induced by a variety of physiological and pathological factors [28]. Previous studies found that Gal-1 inhibited the function of many inflammatory cells, as well as the migration of inflammatory cells to the inflammatory site. Zonon and colleagues found [29] that Gal-1 relieved endotoxin-induced uveitis symptoms by reducing the release of proinflammatory cytokines and inhibiting leukocyte migration. Gal-1 also induced T cell apoptosis. Previous study demonstrated that, in Gal-1 knockout and collagen II-induced arthritis model, the mice were more prone to arthritis after knocking out of Gal-1 gene, and the degree of inflammation was significantly higher than that in wild-type mice [30]. In the autoimmune encephalitis animal model and the ischemic brain injury, administration of recombinant Gal-1 improved the disease symptoms or prevented the occurrence of disease [31]. These studies suggested that Gal-1 had a strong immunosuppressive effect.

This current study showed that, after coculturing MSCs with DCs, the level of Gal-1 in the culture supernatants was increased with increase in the ratio of MSCs to DCs. Immunofluorescence showed that the expression of Gal-1 on the surface of DCs was enhanced, and the expression of Gal-1 protein in DC cells was also significantly increased compared with that in DC only group, whereas the expression of costimulatory molecules on the surface of DCs was decreased, and the effect of DCs on T cell proliferation was attenuated. Thiodigalactoside (TDG) is a nonmetabolizable disaccharide and known as a selective inhibitor of Gal-1 [32]. After adding TDG to MSC + DC group, contradictory results were obtained, suggesting that Gal-1 played an important role during this process. In this study, MSCs were separated from DCs by Transwell chambers, and hence the two types of cells were not in direct contact. Therefore, it was proposed that MSCs secreted Gal-1 to play their immune tolerance role in DCs and to make the T cells incompetent. Therefore, our study indicated that the Gal-1 secreted from MSCs could upregulate the expression of Gal-1 on DCs, which is the first reported so far.

The present study further investigated the mechanism through which Gal-1 was involved in the immunoregulation of DCs. Eukaryotic cells are widely known to express MAPK, which plays a key role in gene expression regulation and cytoplasmic activity [33]. The known MAPK signal transduction pathways that play important roles in DC differentiation and regulation of cytokine secretion mainly include ERK1/2, p38 MAPK, and JNK. This study found that, after adding MSCs or Gal-1 to DCs, ERK phosphorylation levels in DCs were significantly higher than those in DC only group, whereas the p-38 MAPK phosphorylation levels were significantly reduced; contradictory results were obtained on adding TDG. This novel study demonstrated that Gal-1 might inhibit DCs by activating the ERK pathway and inhibiting the P38 MAPK signaling pathway in DCs. Furthermore, the results showed that JNK phosphorylation in DCs was not significantly altered by adding MSCs or recombinant Gal-1 protein, suggesting that Gal-1 did not regulate DCs via the JNK pathway.

The present study also found that, after adding MSCs or Gal-1, the IL-10 and IL-12 levels in the supernatants of the DC group were significantly higher than those in DC only group, whereas contradictory results were obtained after adding TDG, suggesting that the increase in IL-10 and IL-12 levels in the supernatants was positively correlated with Gal-1. IL-10 and IL-12 are two important cytokines secreted by immature DCs. IL-10 limits the intensity of immune response. IL-12 stimulates natural killer cells during the body's immune response to secrete IFN-gamma, which in turn promotes DCs to secrete IL-12, thus forming positive feedback. Such positive feedback promotes differentiation of Th0 toward Thl cells. This microenvironment is beneficial for the body to clear pathogenic microorganisms during the antigenic response [34] and meanwhile avoids excessive immune responses that lead to allergic diseases, thus regulating and maintaining homeostasis of body's immune system. Cedeno et al. also found [35] that Gal-1 promoted the secretion of anti-inflammatory factor IL-10, which inhibited the proliferation of T cells, thereby inhibiting T cell-mediated immune responses, which was consistent with the present findings.

In summary, MSCs have many advantages for clinical treatment: they have a variety of sources, are easily available, have a strong potential of differentiation, and possess characteristics such as easy isolation and culture expansion, stable genetic background, and no immune rejection after implantation. Therefore, MSCs are widely used in fields such as cell replacement therapy, seed source of tissue engineering, and gene therapy. At present, the regulation of MSCs on DCs through secretion of Gal-1 and its mechanism has been rarely explored. The present study indicated Gal-1 secreted from MSCs upregulated the expression of Gal-1 on DCs and stimulated formation of tolerance immunophenotype on DCs, where the underlying mechanism was regulation of MAPK signaling pathway in DCs, thereby inhibiting the function of DCs.
